# Delayed Hypertension Diagnosis and Its Association With Cardiovascular Treatment and Outcomes

**DOI:** 10.1001/jamanetworkopen.2025.20498

**Published:** 2025-07-14

**Authors:** Yuan Lu, John E. Brush, Chungsoo Kim, Yuntian Liu, Xin Xin, Chenxi Huang, Mitsuaki Sawano, Patrick Young, Jacob McPadden, Mark Anderson, John S. Burrows, Jordan R. Asher, Harlan M. Krumholz

**Affiliations:** 1 Center for Outcomes Research and Evaluation, Yale New Haven Hospital, New Haven, Connecticut; 2 Section of Cardiovascular Medicine, Department of Internal Medicine, Yale School of Medicine, New Haven, Connecticut; 3 Sentara Health Research Center, Sentara Health, Virginia Beach, Virginia; 4 Macon & Joan Brock Virginia Health Sciences at Old Dominion University, Norfolk, Virginia; 5 Yale School of Medicine, New Haven, Connecticut; 6 Department of Health Policy and Management, Yale School of Public Health, New Haven, Connecticut

## Abstract

**Importance:**

Hypertension is a major risk factor for cardiovascular disease, yet delays in diagnosis may limit timely treatment initiation and increase cardiovascular risk.

**Objectives:**

To examine the timing of clinical hypertension diagnosis, its association with antihypertensive medication prescribing, and its association with long-term cardiovascular outcomes.

**Design, Setting, and Participants:**

This retrospective cohort study analyzed electronic health record (EHR) data from an integrated health care system. Adults aged 18 to 85 years with at least 2 outpatient blood pressure (BP) readings of 140/90 mm Hg or more recorded at least 30 days apart from January 1, 2010, to December 31, 2021, were included (meeting BP-based criteria for hypertension as recorded in the EHR; ie, a “computed hypertension diagnosis”). Data were analyzed from January to November 2023.

**Exposures:**

The timing of clinical hypertension diagnosis (the formal recorded diagnosis by a clinician) relative to the second elevated BP measurement was categorized as (1) preexisting diagnosis; (2) diagnosed between the first and second BP elevations (reference group); (3) diagnosed after the second BP elevation, subdivided into 1 to 90 days, 91 to 365 days, or more than 365 days after the second BP elevation; and (4) no recorded diagnosis.

**Main Outcomes and Measures:**

The primary outcome was antihypertensive medication prescription within 30 days of diagnosis. The secondary outcome was the 5-year composite risk of myocardial infarction, ischemic stroke, or heart failure hospitalization. A multivariable Cox proportional hazards regression model estimated hazard ratios (HRs) for cardiovascular outcomes, adjusting for demographics, BP, and comorbidities.

**Results:**

Among 311 743 patients with a computed hypertension diagnosis, 14.6% received a diagnosis after the second BP elevation (mean [SD] age 57.9 [13.1] years; 53.3% women; 69.6% non-Hispanic White, 24.9% non-Hispanic Black, 2.4% Hispanic or Latino, and 1.7% non-Hispanic Asian). Delayed diagnosis was associated with lower antihypertensive medication prescription rates (30.6% vs 75.2%; *P* < .001) and increased cardiovascular risk over 5 years (delay of 1-90 days: HR, 1.04 [95% CI, 0.95-1.13]; delay of 91-365 days: HR, 1.11 [95% CI, 1.04-1.19]; and delay of >365 days: HR, 1.29 [95% CI, 1.23-1.36]). Greater delay in clinical diagnosis of hypertension was associated with younger age (45-64 years: median delay, 17.5 months [IQR, 6.1-34.6 months] vs ≥75 years: median delay, 13.4 months [IQR, 4.7-28.2 months]; *P* < .001), female sex (median delay, 16.6 months [IQR, 5.8-33.7 months] vs male sex: median delay, 16.1 months [IQR, 5.7-33.1 months]; *P* < .001), and non-Hispanic Asian or non-Hispanic Black race (non-Hispanic Asian: median delay, 18.5 months [IQR, 6.9-34.0 months]; non-Hispanic Black: median delay, 17.2 months [IQR, 5.8-34.9 months]; vs non-Hispanic White: median delay, 16.3 months [IQR, 5.9-33.3 months]).

**Conclusions and Relevance:**

This study suggests that delays in hypertension diagnosis were common and significantly associated with delays in treatment initiation and adverse cardiovascular outcomes, underscoring the need for earlier identification and intervention.

## Key Points

**Question:**

How is the timing of clinical hypertension diagnosis associated with antihypertensive treatment initiation and long-term cardiovascular outcomes?

**Findings:**

In this cohort study of 311 743 patients who met blood pressure–based criteria for hypertension as recorded in the electronic health record, 14.6% received a diagnosis after the second elevated blood pressure reading. Delayed diagnosis was associated with lower antihypertensive medication prescription rates and a higher risk of cardiovascular events over 5 years, with hazard ratios of 1.04 for delays of 1 to 90 days, 1.11 for delays of 91 to 365 days, and 1.29 for delays over 365 days.

**Meaning:**

This study suggests that delayed hypertension diagnosis is common and is associated with lower treatment rates and higher long-term cardiovascular risk, underscoring the need for earlier identification and intervention.

## Teaser

This cohort study uses electronic health record data from a large health system to examine the timing of clinical hypertension diagnosis and its association with antihypertensive medication prescribing and long-term cardiovascular outcomes.

## Introduction

Hypertension affects nearly 50% of individuals in the US, and its persistent elevation can lead to severe health complications.^1,2^ Evidence-based guidelines emphasize the importance of early detection and treatment to mitigate the cumulative risk of adverse outcomes over time.^3^ Timely diagnosis is critical for initiating effective treatment, but the association of diagnostic delay with cardiovascular outcomes is understudied.

Several factors can be associated with delays in diagnosing and treating hypertension. Clinicians may be unaware of all blood pressure (BP) measurements obtained across various care settings or may overlook elevated readings during busy clinical workflows.^4^ For example, patients may have elevated BP at urgent care or specialty visits, but these readings might not be flagged in primary care electronic health record (EHR) systems. In addition, inconsistencies across recommendations from professional societies may contribute to variability in clinical practice.^5^

Prior studies using EHR data indicate that approximately 30% of patients with persistently elevated BP lack a formal hypertension diagnosis or documented treatment.^6,7,8,9,10^ However, these studies have not quantified the extent of diagnostic delays or examined their association with downstream outcomes, such as antihypertensive medication initiation and long-term cardiovascular risk, across diverse populations.

The digital transformation of health care presents a key opportunity to bridge the gap between elevated BP readings recorded in the EHR and the formal clinical diagnosis of hypertension. Blood pressure, as a quantifiable and easily interpretable metric, is well-suited for computational analysis. Moreover, EHRs aggregate data from multiple care settings, offering a comprehensive foundation for improving hypertension care delivery. By developing an operational definition for a computed hypertension diagnosis and integrating prompts or reminders into clinical workflows, health systems can leverage EHRs to facilitate more timely and accurate hypertension diagnoses, ultimately enhancing patient outcomes.

Despite prior research highlighting underdiagnosis of hypertension, the extent of diagnostic delay and its consequences for cardiovascular outcomes remain poorly quantified. This study examines how the timing of hypertension diagnosis is associated with treatment initiation and cardiovascular risk, leveraging EHR data from a large regional health system. Our findings aim to provide actionable insights to support earlier intervention and improve patient outcomes.

## Methods

### Data Source

We performed a retrospective cohort analysis of EHR data from a large nonprofit integrated health care system with 12 hospitals, 566 outpatient sites, and over 1300 physicians and other clinical health professionals in Virginia and northeastern North Carolina. The system adopted a secure, centralized EHR platform developed by Epic in 2007 to manage clinical and administrative claims data. In 2021, these data were transformed into the Observational Medical Outcomes Partnership (OMOP) common data model, version 5.3, to support standardized analysis and outcomes research.^11^ The study was approved by the institutional review board at Eastern Virginia Medical School. The requirement for informed consent was waived because the study used deidentified electronic health record data and posed minimal risk to participants. This study followed the Strengthening the Reporting of Observational Studies in Epidemiology (STROBE) guideline.^12^

### Cohort Definition and BP Measurements

The study population included adult patients aged 18 to 85 years who had at least 2 outpatient BP readings recorded on different dates between January 1, 2010, and December 31, 2021. Patients older than 85 years were excluded to minimize potential biases associated with frailty, comorbidities, and atypical BP patterns that might not align with standard hypertension management guidelines. An extended timeframe from January 1, 2008, to December 31, 2022, was used to examine records of visits and diagnoses before and after the BP recording dates. Patients who were pregnant or undergoing dialysis were excluded from the analysis.

Blood pressure readings were analyzed at the visit level: if more than 1 BP reading was taken during a visit, the first reading was disregarded, and the mean of the remaining readings was used as the visit BP measurement. For patients with multiple outpatient visits on the same date, the mean of all visit BP measurements was calculated and recorded as the BP measurement for that day. To reduce the undue association of transiently elevated BP from acute medical conditions, we analyzed only outpatient BP values, and excluded BP readings from inpatient, emergency department, or ambulatory surgery center visits.^7,13^ Race and ethnicity were self-reported and extracted from the EHR, then mapped to the OMOP common data model, which assigns each patient to a single category: Hispanic or Latino (regardless of race), non-Hispanic Asian, non-Hispanic Black, non-Hispanic White, or other or unknown (which includes unspecified or unlisted identities). Race and ethnicity were included to evaluate potential disparities in the timing of hypertension diagnosis and associated outcomes.

### Computed Diagnosis and Clinical Diagnosis of Hypertension

We defined a computed hypertension diagnosis as having at least 2 consecutive elevated BP measurements recorded within a 2-year period, with at least 30 days between readings. Elevated BP was defined as a systolic BP (SBP) of 140 mm Hg or higher or a diastolic BP (DBP) of 90 mm Hg or higher to align with the coding practices during the study period. A clinical hypertension diagnosis was defined as the recorded presence of a diagnosis code for hypertension associated with at least 1 outpatient visit during the study period. The complete list of hypertension diagnoses and their corresponding OMOP concept identification numbers are in eTable 1 in Supplement 1.

The timing of clinical hypertension diagnosis was assessed and categorized into the following categories: “Diagnosed before first BP elevation” (indicating a preexisting history of hypertension), “Diagnosed between first and second BP elevations,” and “Diagnosed after second BP elevation,” which was further subcategorized into diagnoses made within 1 and 90 days, 91 and 365 days, or more than 365 days after the second BP elevation. We considered a clinical diagnosis of hypertension recorded after the second elevated BP reading as a delayed diagnosis of hypertension. Patients who received a hypertension diagnosis more than 5 years after the second elevated BP reading and cases with no recorded clinical hypertension diagnosis during the study period were classified as “No clinical diagnosis.”

### Antihypertensive Medication Prescriptions

Medication prescription orders for each outpatient visit were extracted from the EHR. Active prescription was defined when the prescription order start date was between 180 days prior to the second BP elevation and 30 days after the second BP elevation. First-line antihypertensive medications were defined as thiazide or thiazide-like diuretics, angiotensin-converting enzyme inhibitors, angiotensin receptor blockers, or dihydropyridine or nondihydropyridine calcium channel blockers according to the 2017 American Heart Association–American College of Cardiology hypertension guideline.^10^ Second-line antihypertensive medications, also based on this guideline, included beta-blockers, alpha-blockers, and other diuretics (full list of medications in eTable 2 in Supplement 1).

### Adverse Cardiovascular Outcomes

The primary outcome was a composite of adverse cardiovascular events, including hospitalization for acute myocardial infarction, hospitalization for heart failure, and clinically diagnosed ischemic stroke during an inpatient or emergency department visit. Secondary outcomes were the individual components of the composite outcome. All study outcomes were restricted to new conditions that were recorded during emergency department or inpatient visits, assessed over a maximum 5-year time frame. Cardiovascular outcomes were defined using standard OMOP phenotype algorithms that have been previously validated and used in large-scale studies^14,15^ (associated OMOP concept identification numbers are listed in eTable 3 in Supplement 1).

### Statistical Analysis

Statistical analysis was conducted from January to November 2023. We used descriptive statistics to characterize patients with a computed diagnosis of hypertension. Continuous variables were compared using the *t* test and Kruskal-Wallis test for comparisons of means or medians and 1-way analysis of variance for comparisons across more than 2 groups, as appropriate. Categorical variables were analyzed using the χ^2^ test. Trend analysis for categorical variables was conducted using the Cochran-Armitage trend test. Baseline characteristics were assessed as of the date of the second elevated BP measurement, which served as the index date for cohort entry. We then assessed the number of outpatient visits per patient stratified by diagnostic delay category.

We also examined the association of the timing of a clinical hypertension diagnosis with cardiovascular outcomes using survival analysis. The maximum follow-up date was defined as 5 years after the date of clinical diagnosis of hypertension or the last date of the database for patients who did not experience cardiovascular events during the study period. A Kaplan-Meier survival curve was generated using the age-adjusted Cox proportional hazards regression model. We assessed the proportional hazards assumption for all Cox proportional hazards regression models using Schoenfeld residuals on different delayed groups and the assumption was satisfied for all models included in the analysis. The hazard ratio (HR) for adverse cardiovascular outcome was estimated for each group using a multivariable Cox proportional hazards regression model. Time zero was set at the date of clinical diagnosis of hypertension and the reference group was patients diagnosed between the first and second BP elevations (eFigure 1 in Supplement 1). The Cox proportional hazards regression model was adjusted for demographics (age, race and ethnicity, sex), BP, and comorbidities at or before the time of clinical diagnosis of hypertension.

All statistical tests were 2-sided, and results were deemed statistically significant at *P* < .05. Data analysis was performed using R, version 4.2.3 (R Project for Statistical Computing).

## Results

### Patient Characteristics and Health Care Use

Among 311 743 patients with a computed hypertension diagnosis, 45 454 (14.6%) were diagnosed after the second BP elevation (mean [SD] age; 57.9 [13.1]; 24 244 women [53.3%] and 21 210 men [46.7%]; 1103 Hispanic patients [2.4%], 788 non-Hispanic Asian patients [1.7%], 11 310 non-Hispanic Black patients [24.9%], 31 614 non-Hispanic White patients [69.6%], and 639 patients of other or unknown races [1.4%]; Table 1). The study flowchart is provided in eFigure 2 in Supplement 1. The demographics of patients diagnosed between the first and second BP elevations, those diagnosed after the second BP elevation, and those with no clinical diagnosis are shown in Table 1. Overall, delay in clinical diagnosis of hypertension was associated with younger age (45-64 years: median delay, 17.5 months [IQR, 6.1-34.6 months] vs ≥75 years: median delay, 13.4 months [IQR, 4.7-28.2 months]; *P* < .001), female sex (median delay, 16.6 months [IQR, 5.8-33.7 months] vs male sex: median delay, 16.1 months [IQR, 5.7-33.1 months]; *P* < .001), and non-Hispanic Asian or non-Hispanic Black race (non-Hispanic Asian: median delay, 18.5 months [IQR, 6.9-34.0 months]; non-Hispanic Black: median delay, 17.2 months [IQR, 5.8-34.9 months]; vs non-Hispanic White: median delay, 16.3 months [IQR, 5.9-33.3 months]) (eTable 4 in Supplement 1). The median number of outpatient BP measurements per patient during the study period was 16.0 (IQR, 8.0-30.0). Mean (SD) SBP and DBP values varied across groups, with the highest SBP observed among patients who received a diagnosis within 1 to 90 days after the second BP elevation (SBP, 149.7 [13.3] mm Hg and DBP, 86.7 [9.8] mm Hg). In comparison, mean (SD) BP values were slightly lower among those diagnosed between the first and second BP elevations (SBP, 148.9 [13.6] mm Hg and DBP, 86.2 [10.1] mm Hg) and among those diagnosed after longer delays (91-365 days: SBP, 147.4 [12.3] mm Hg and DBP, 85.4 [9.4] mm Hg; and >365 days: SBP, 146.7 [12.2] mm Hg and DBP, 84.7 [9.2] mm Hg) (Table 1). Patients without a clinical hypertension diagnosis had the lowest mean (SD) BP values (SBP, 146.3 [11.8] mm Hg and DBP, 83.8 [9.4] mm Hg) and fewer comorbidities compared with those with a diagnosis of hypertension. Given these differences, this group was not the focus of our primary analysis, as our objective was to evaluate the association of diagnostic delay among patients who ultimately received a hypertension diagnosis.

**Table 1.** Demographic Characteristics and Age-Adjusted Mean Blood Pressure of Patients With a Computed Hypertension Diagnosis

Characteristic^a^	Diagnosed between first and second BP elevations (n = 65 269)	Diagnosed after second BP elevation (n = 45 454)			No clinical diagnosis (n = 94 956)	*P* value^b^
		Days 1-90 (n = 7200)	Days 91-365 (n = 11 394)	After day 365 (n = 26 860)		
Sex, No. (%)						
Female	32 775 (50.2)	3784 (52.6)	5931 (52.1)	14 529 (54.1)	50 283 (53.0)	<.001
Male	32 494 (49.8)	3416 (47.4)	5463 (47.9)	12 331 (45.9)	44 667 (47.0)	
Missing	0	0	0	0	6 (0.006)	
Race and ethnicity, No. (%)						
Hispanic or Latino	1602 (2.5)	173 (2.4)	283 (2.5)	647 (2.4)	2640 (2.8)	<.001
Non-Hispanic Asian	1217 (1.9)	124 (1.7)	179 (1.6)	485 (1.8)	1346 (1.4)	
Non-Hispanic Black	20 015 (30.7)	1775 (24.7)	2795 (24.5)	6740 (25.1)	19 384 (20.4)	
Non-Hispanic White	41 282 (63.2)	5009 (69.6)	7968 (69.9)	18 637 (69.4)	69 151 (72.8)	
Other or unknown^c^	1153 (1.8)	119 (1.7)	169 (1.5)	351 (1.3)	2435 (2.6)	
Age, mean (SD), y	59.4 (13.4)	58.4 (13.4)	58.3 (13.4)	57.6 (12.9)	55.7 (15.9)	<.001
Age group, y, No. (%)						
18-44	9420 (14.4)	1157 (16.1)	1834 (16.1)	4337 (16.1)	23 806 (25.1)	<.001
45-64	30 589 (46.9)	3473 (48.2)	5495 (48.2)	13 685 (50.9)	40 881 (43.1)	
65-74	16 265 (24.9)	1704 (23.7)	2685 (23.6)	6297 (23.4)	17 925 (18.9)	
≥75	8995 (13.8)	866 (12.0)	1380 (12.1)	2541 (9.5)	12 344 (13.0)	
SBP, mean (SD), mm Hg^d^	148.9 (13.6)	149.7 (13.3)	147.4 (12.3)	146.7 (12.2)	146.3 (11.8)	<.001
DBP, mean (SD), mm Hg^d^	86.2 (10.1)	86.7 (9.8)	85.4 (9.4)	84.7 (9.2)	83.8 (9.4)	<.001
Comorbidities, No. (%)						
Alzheimer disease	189 (0.3)	12 (0.2)	37 (0.3)	51 (0.2)	242 (0.3)	.03
Chronic liver disease	825 (1.3)	103 (1.4)	143 (1.3)	300 (1.1)	732 (0.8)	.37
Chronic kidney disease	3290 (5.0)	354 (4.9)	502 (4.4)	766 (2.9)	1297 (1.4)	<.001
COPD	4752 (7.3)	551 (7.7)	956 (8.4)	1815 (6.8)	3516 (3.7)	.87
Dyslipidemia	40 410 (61.9)	3562 (49.5)	5874 (51.6)	13 121 (48.8)	20 644 (21.7)	<.001
Type 2 diabetes	17 837 (27.3)	1492 (20.7)	2484 (21.8)	5092 (19.0)	6019 (6.3)	<.001
Malignant neoplasm	8822 (13.5)	1131 (15.7)	1719 (15.1)	4300 (16.0)	9305 (9.8)	<.001

Abbreviations: BP, blood pressure; COPD, chronic obstructive pulmonary disease; DBP, diastolic blood pressure; SBP, systolic blood pressure.

^a^ Baseline characteristics were assessed as of the date of the second elevated BP measurement, which served as the index date for cohort entry. A delayed clinical diagnosis was defined as hypertension diagnosed after the second elevated BP measurement.

^b^ *P* value comparing patients who received a diagnosis between the first and second BP elevations and patients who received a diagnosis after the second BP elevation.

^c^ The other or unknown category included individuals who either selected Other as their racial or ethnic identity or did not provide this information.

^d^ Blood pressures were age adjusted.

The number of outpatient and primary care visits across diagnostic timing groups are presented in Table 2. Before the second elevated BP reading, the median number of outpatient and primary care visits was similar between the early-diagnosis and delayed-diagnosis groups (8 [IQR, 3-17] vs 7 [IQR, 3-15] visits). After the second elevated BP reading, substantial health care use persisted among patients with delayed diagnoses. The median number of outpatient visits increased with longer diagnostic delays, ranging from 2 (IQR, 1-3) among those who received a diagnosis within 1 to 90 days to 4 (IQR, 2-8) among those who received a diagnosis within 91 to 365 days and 14 (IQR, 6-28) among those who received a diagnosis more than 365 days after the second BP elevation. A similar pattern was observed for primary care visits.

**Table 2.** Outpatient Visits by Diagnostic Delay

Group	Median (IQR)		
	Any visits, No.	Outpatient visits, No.	Primary care visits, No.^a^
**Health care visits between the first and second elevated BP readings**			
Diagnosed between first and second BP elevations	8 (3-17)	3 (1-6)	4 (2-7)
Diagnosed days 1-90 after the second BP elevation	7 (3-15)	3 (1-5)	3 (2-6)
Diagnosed days 91-365 after the second BP elevation	7 (3-15)	3 (1-6)	3 (2-7)
Diagnosed after day 365 after the second BP elevation	7 (3-15)	3 (1-6)	3 (2-7)
No clinical diagnosis	8 (3-17)	3 (1-6)	4 (2-7)
**Health care visits between the second elevated BP and hypertension diagnosis**			
Diagnosed days 1-90 after the second BP elevation	4 (2-8)	2 (1-3)	2 (1-3)
Diagnosed days 91-365 after the second BP elevation	11 (6-22)	4 (2-8)	4 (2-8)
Diagnosed after day 365 after the second BP elevation	43 (21-85)	14 (6-28)	13 (5-29)

Abbreviation: BP, blood pressure.

^a^ Primary care visits included visits to the primary care, family medicine, and internal medicine departments.

### Association of Hypertension Diagnosis Timing With Medication Prescriptions

**Figure 1.**
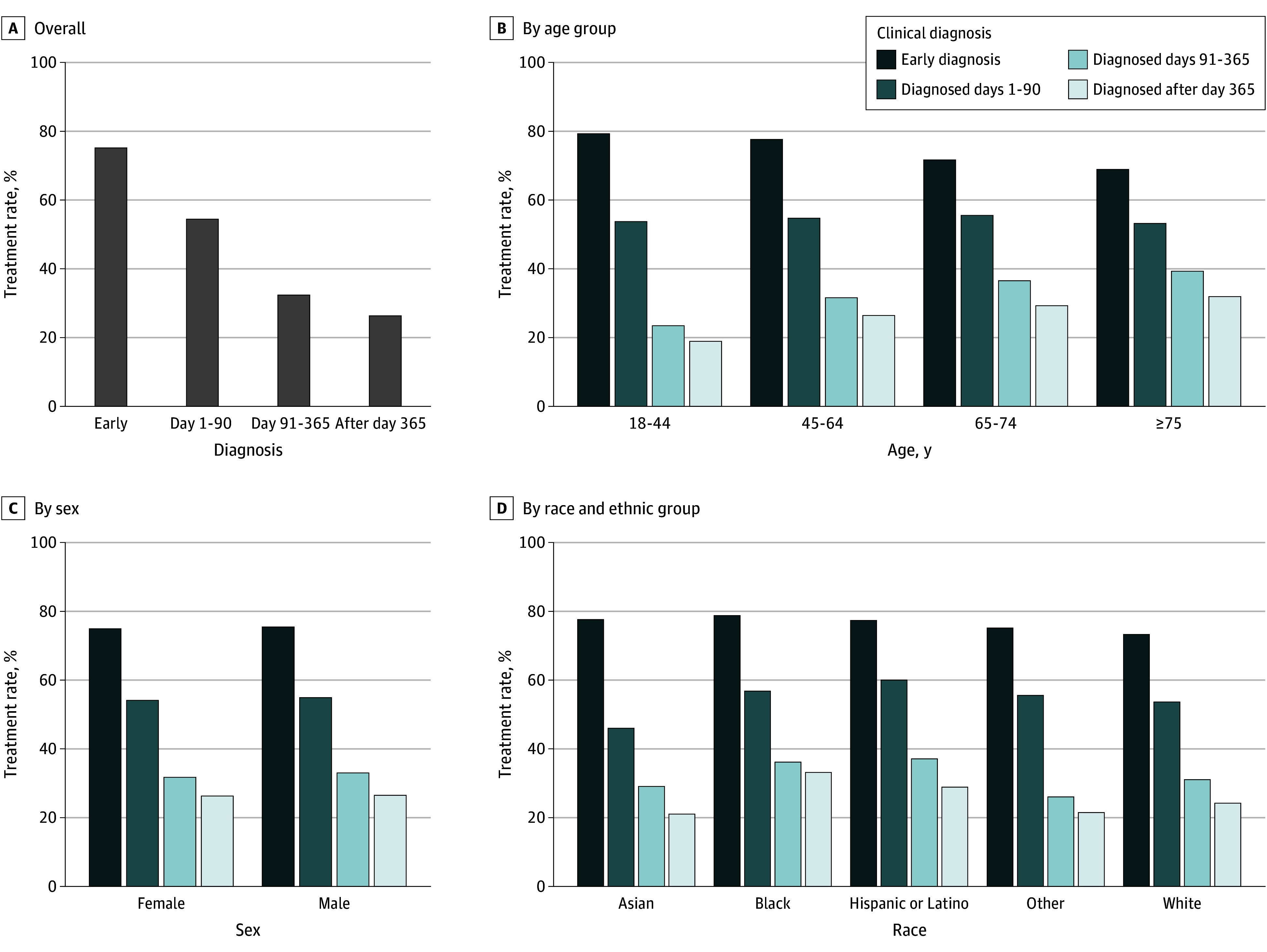
Prescription Rates of Antihypertensive Agents by Timing of Hypertension Diagnosis A, Prescription rates of antihypertensive agents for overall study population. B, Prescription rates of antihypertensive agents for by age group. C, Prescription rates of antihypertensive agents for by sex. D, Prescription rates of antihypertensive agents for by race and ethnicity.

The prescription rate for antihypertensive medications varied significantly based on the timing of hypertension diagnosis. Patients diagnosed between the first and second BP elevations were more likely to receive antihypertensive treatment compared with those diagnosed after the second BP elevation (75.2% vs 30.6% of overall antihypertensive medication prescription rate among patients diagnosed after the second BP elevation; absolute difference, 44.6% [95% CI 44.1%-45.1%]; *P* < .001; χ^2^_1_ = 21731.6). Among patients diagnosed after the second BP elevation, the likelihood of receiving an antihypertensive medication prescription decreased with greater delay, with treatment rates of 54.5% (3924 of 7200) for diagnoses made within 1 to 90 days, 32.4% (3692 of 11 394) for diagnoses made within 90 to 365 days, and 26.4% (7091 of 26 860) for diagnoses made within more than 365 days after the second BP elevation (*P* < .001; χ^2^_2_ = 2048.5; Figure 1). This declining trend in prescription rates was consistent across various subgroups stratified by age groups, sex, and race and ethnicity (Figure 1; eTable 5 in Supplement 1). A sensitivity analysis examining only first-line antihypertensive medications showed a similar pattern of declining prescription rates with longer diagnostic delays (eFigure 3 in Supplement 1).

### Association of Hypertension Diagnosis Timing With Adverse Cardiovascular Outcomes

**Figure 2.**
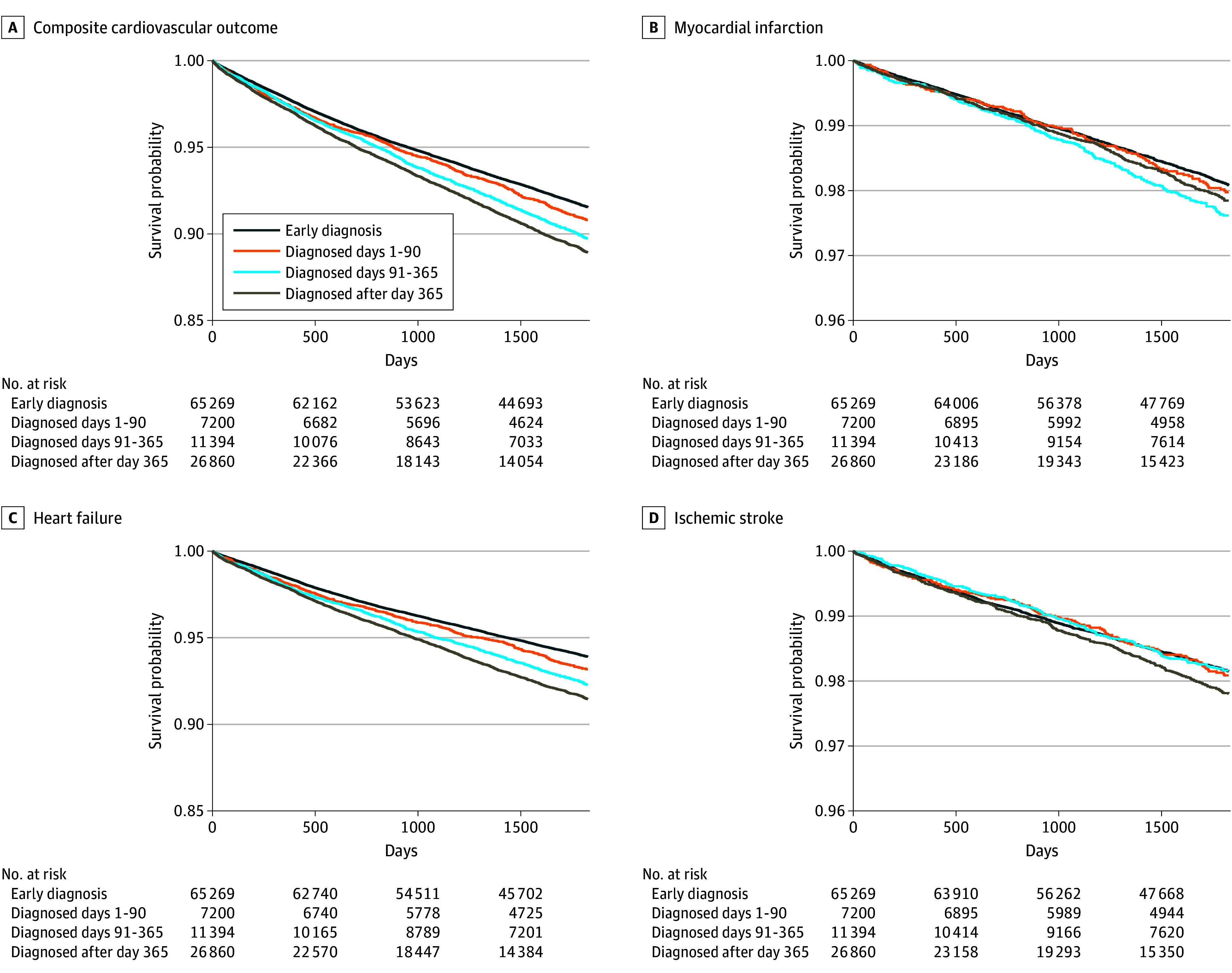
Age-Adjusted Survival Curves for Adverse Cardiovascular Outcomes Over a 5-Year Period A, Composite cardiovascular outcome. B, Myocardial infarction. C, Heart failure. D, Ischemic stroke.

Patients who received a diagnosis of hypertension after the second BP elevation had a significantly higher risk of adverse cardiovascular outcomes over 5 years compared with those who received diagnosis between the first and second BP elevations. The HRs for cardiovascular outcomes increased progressively with longer delays in diagnosis, compared with patients diagnosed between the first and second BP elevations (reference group): HR, 1.04 (95% CI, 0.95-1.13) for diagnoses made within 1 to 90 days; HR, 1.11 (95% CI, 1.04-1.19) for 91 to 365 days; and HR, 1.29 (95% CI, 1.23-1.36) for over 365 days after the second BP elevation, controlling for multiple covariates (Table 3). This pattern was consistent across the 3 specific components of the combined outcome measure. The association was most pronounced for heart failure hospitalizations (HR, 1.03 [95% CI, 0.94-1.13] for diagnoses made within 1-90 days; HR, 1.11 [95% CI, 1.03-1.20] for diagnoses made within 91-365 days; and HR, 1.31 [95% CI, 1.24-1.39] for diagnoses made >365 days after the second BP elevation). For ischemic stroke, the HRs were 1.06 (95% CI, 0.88-1.27) for diagnoses made within 1 to 90 days, 0.98 (95% CI, 0.84-1.15) for diagnoses made within 90 to 365 days, and 1.19 (95% CI, 1.07-1.33) for diagnoses made more than 365 days after the second BP elevation. Figure 2 illustrates the age-adjusted survival curves derived from the Cox proportional hazards regression models, showing clear stratification of cardiovascular event risks by the timing of diagnosis. Subgroup analyses confirmed these findings, with delayed diagnosis consistently associated with higher cardiovascular risk across demographic groups, including age, sex, and non-Hispanic White and non-Hispanic Black patients (eFigure 4 in Supplement 1).

**Table 3.** Hazard Ratios of Temporal Delays in Hypertension Diagnosis With Adverse Cardiovascular Outcomes

Group	Age-adjusted Cox model		Multivariable adjusted Cox model^a^	
	HR (95% CI)	*P* value	HR (95% CI)	*P* value
Diagnosed between first and second BP elevations	1 [Reference]	NA	1 [Reference]	NA
Diagnosed days 1-90 after the second BP elevation	1.09 (1.00-1.18)	.04	1.04 (0.95-1.13)	.41
Diagnosed days 91-365 after the second BP elevation	1.21 (1.14-1.30)	<.001	1.11 (1.04-1.19)	.002
Diagnosed after day 365 after the second BP elevation	1.32 (1.26-1.39)	<.001	1.29 (1.23-1.36)	<.001

Abbreviations: BP, blood pressure; NA, not applicable.

^a^ The following covariates were adjusted in the multivariable Cox proportional hazards regression model: age, sex, race and ethnicity, BP (systolic and diastolic BP), and comorbidities (Alzheimer disease, chronic kidney disease, chronic liver disease, cancer, type 2 diabetes, dyslipidemia, peripheral artery disease, myocardial infarction, stroke, and heart failure).

## Discussion

This study demonstrates that delays in hypertension diagnosis are common and are associated with progressively worse patient outcomes. Delayed diagnosis was associated with a reduced likelihood of antihypertensive medication treatment and significantly increased cardiovascular risk, with the highest risk among those diagnosed more than 1 year after documented BP elevations.

Previous studies have highlighted the underdiagnosis of hypertension,^6,7,8,9,10^ but the extent of diagnostic delays and their consequences for cardiovascular outcomes have remained less well quantified. Our findings extend prior work by demonstrating that not only are delays in hypertension diagnosis frequent but they also follow a graded association with both lower antihypertensive treatment initiation and increased long-term cardiovascular risk. Patients diagnosed more than 1 year after the second elevated BP measurement had a nearly 30% higher risk of cardiovascular events compared with those diagnosed earlier. These findings align with prior evidence, including a large population-based study in the UK showing that delayed initiation of antihypertensive therapy is associated with increased risk of major adverse cardiovascular events.^16^ Our findings highlight the clinical significance of delayed hypertension recognition and the potential association of earlier intervention with mitigation of cardiovascular risk.

Although one possible explanation for delayed diagnosis is infrequent health care engagement, our findings suggest otherwise. Patients with delayed hypertension diagnoses had a similar number of outpatient and primary care visits before their second elevated BP reading compared with those diagnosed earlier. Moreover, many of these patients continued to engage with the health care system after the second BP elevation, with longer diagnostic delays associated with a higher number of outpatient and primary care visits. These findings suggest that delays were primarily due to missed clinical opportunities rather than patient disengagement from care.

However, alternative explanations should also be considered. Clinicians may hesitate to diagnose hypertension based on a single elevated BP measurement, particularly when concerned about white-coat hypertension (ie, uncontrolled clinic-measured BP, with either home or ambulatory readings at goal) or transient BP elevations.^17^ Current guidelines emphasize confirming sustained hypertension before initiating treatment^3^ and recommend lifestyle modification for patients with stage 1 hypertension and low cardiovascular risk prior to starting medication. Such a guideline-concordant approach may explain some delays and could be misinterpreted as therapeutic inertia. However, the consistently lower rates of antihypertensive medication prescriptions among patients with delayed diagnoses suggest that clinical caution alone does not fully account for the observed delays. Finally, we observed longer diagnostic delays among younger adults, women, and non-Hispanic Black and non-Hispanic Asian patients, raising the possibility that implicit bias,^3^ differences in symptom presentation, or variability in clinician-patient interactions^18^ may be associated with disparities in diagnostic timing.

The persistence of delayed diagnoses despite frequent health care interactions highlights the need for patient-specific interventions to improve recognition of hypertension. Current clinical workflows often rely on clinician recall or manual recognition of elevated BP readings, which may be associated with oversight, particularly in busy outpatient settings.^19^ EHR-based decision support tools could help address these challenges by systematically identifying patients who meet hypertension criteria and prompting clinicians to take action.^20^ Expanding the role of nonphysician health care professionals offers another opportunity to improve hypertension detection.^21^ Pharmacists, as integral members of team-based care models, can contribute significantly to BP screening, medication initiation and titration, follow-up monitoring, and patient education, especially in outpatient and community settings. Similarly, nurses can contribute to BP rechecks, patient education, and referral for confirmatory testing. Embedding team-based care models within primary care practices may help distribute the workload and ensure that patients meeting hypertension criteria receive timely evaluation and diagnosis.

Although reducing diagnostic delays is a critical goal, efforts to increase detection of hypertension should be balanced against the risk of overdiagnosis, as misclassifying transient BP elevations as sustained hypertension may lead to overtreatment. More frequent BP reassessment and ambulatory BP monitoring could help ensure that true hypertension is distinguished from temporary elevations.^22^ In addition, increased diagnostic sensitivity could contribute to greater workload for primary care physicians, highlighting the need for structured systems to manage flagged patients efficiently. Integrating decision support tools with robust, interdisciplinary care teams—including physicians, nurses, and pharmacists—can improve the accuracy, timeliness, and overall quality of hypertension diagnosis and management.

### Limitations

These findings should be interpreted in the context of several limitations. First, this study was conducted within a regional health care system, which may limit generalizability to other settings with different patient populations and care models. Second, we were unable to assess home BP monitoring or ambulatory BP data, which could influence clinician decision-making. Third, reliance on EHR-documented diagnoses may underestimate hypertension recognition if informal diagnoses were made without corresponding codes. We were also unable to assess cardiovascular mortality due to incomplete cause-of-death data, which may lead to underestimation of the association of diagnostic delay with outcomes. Residual confounding is possible, as unmeasured factors—such as social determinants of health, patient preferences, or clinician judgment—may be associated with both diagnosis timing and outcomes. Although EHRs offer detailed clinical data, they do not capture the full context of patient–clinician decision-making. Future qualitative studies could help elucidate behavioral and systemic factors associated with diagnostic delays. Fourth, our use of a threshold BP of 140/90 mm Hg or higher reflects prevailing clinical practice during the study period. However, newer US guidelines recommend a lower threshold of 130/80 mm Hg or higher for high-risk individuals.^3^ Applying these updated criteria in future research may reveal a greater burden of undiagnosed hypertension and inform targeted strategies to promote earlier diagnosis and treatment.

## Conclusions

In this cohort study of 311 743 adults with a computed hypertension diagnosis, delays in hypertension diagnosis were common and were associated with lower antihypertensive treatment rates and higher long-term cardiovascular risk. Interventions leveraging EHR systems may help facilitate earlier recognition and treatment of hypertension, potentially reducing cardiovascular risk.

## Notes

Supplement 1. **eTable 1.** Hypertension Coding Terms With Corresponding OMOP Concept IDs **eTable 2.** Antihypertensive Drug Classes With Corresponding Drug Names and OMOP Concept IDs **eTable 3.** Adverse Cardiovascular Outcomes With Corresponding OMOP Concept IDs **eTable 4.** Temporal Delay in Hypertension Diagnosis by Demographic Subgroups **eTable 5.** Results of Cochran-Armitage Trend Test on Antihypertensive Medication Use According to Different Diagnosis Timing in Subgroups **eFigure 1.** Diagram Illustrating Categories of Diagnostic Timing **eFigure 2.** Study Flow Chart **eFigure 3.** Prescription Rates of First-Line Antihypertensive Agents by Timing of Hypertension Diagnosis **eFigure 4.** Age-Adjusted Survival Curves for Adverse Cardiovascular Outcomes Over a 5-Year Period by Age, Sex, Race, and Ethnicity

Supplement 2.
Data Sharing Statement
