# Supplementary material for: Delayed Hypertension Diagnosis and Its Association With Cardiovascular Treatment and Outcomes
Source: JAMA Netw Open. 2025 Jul 14;8(7):e2520498. doi: 10.1001/jamanetworkopen.2025.20498 (PMC12261005; doi:10.1001/jamanetworkopen.2025.20498)
Supplement: Supplement 1. — eTable 1. Hypertension Coding Terms With Corresponding OMOP Concept IDs eTable 2. Antihypertensive Drug Classes With Corresponding Drug Names and OMOP Concept IDs eTable 3. Adverse Cardiovascular Outcomes With Corresponding OMOP Concept IDs eTable 4. Temporal Delay in Hypertension Diagnosis by Demographic Subgroups eTable 5. Results of Cochran-Armitage Trend Test on Antihypertensive Medication Use According to Different Diagnosis Timing in Subgroups eFigure 1. Diagram Illustrating Categories of Diagnostic Timing eFigure 2. Study Flow Chart eFigure 3. Prescription Rates of First-Line Antihypertensive Agents by Timing of Hypertension Diagnosis eFigure 4. Age-Adjusted Survival Curves for Adverse Cardiovascular Outcomes Over a 5-Year Period by Age, Sex, Race, and Ethnicity [file jamanetwopen-e2520498-s001.pdf]

Supplementary Online Content

Lu Y, Brush JE, Kim C, et al. Delayed hypertension diagnosis and its association with cardiovascular treatment and outcomes. *JAMA Netw Open.* 2025;8(7):e2520498. doi:10.1001/jamanetworkopen.2025.20498

eTable 1. Hypertension Coding Terms With Corresponding OMOP Concept IDs
eTable 2. Antihypertensive Drug Classes With Corresponding Drug Names and OMOP Concept IDs
eTable 3. Adverse Cardiovascular Outcomes With Corresponding OMOP Concept IDs
eTable 4. Temporal Delay in Hypertension Diagnosis by Demographic Subgroups
eTable 5. Results of Cochran-Armitage Trend Test on Antihypertensive Medication Use According to Different Diagnosis Timing in Subgroups
eFigure 1. Diagram Illustrating Categories of Diagnostic Timing
eFigure 2. Study Flow Chart
eFigure 3. Prescription Rates of First-Line Antihypertensive Agents by Timing of Hypertension Diagnosis
eFigure 4. Age-Adjusted Survival Curves for Adverse Cardiovascular Outcomes Over a 5-Year Period by Age, Sex, Race, and Ethnicity

This supplementary material has been provided by the authors to give readers additional information about their work.

© 2025 Lu Y et al. *JAMA Network Open.*

eTable 1. Hypertension Coding Terms With Corresponding OMOP Concept IDs

**Term**	**Concept ID**
Essential hypertension	320128
Hypertension secondary to endocrine disorder	4110948
Secondary hypertension	319826
Renovascular hypertension	317895
Hypertensive urgency	40481896
Hypertensive emergency	43020424
Renal hypertension	443771
Hypertensive crisis	45768449
Malignant essential hypertension	317898

© 2025 Lu Y et al. *JAMA Network Open.*

eTable 2. Antihypertensive Drug Classes With Corresponding Drug Names and OMOP Concept IDs

**Class**	**Drug Name (Concept ID)**
Thiazide-like diuretics	Chlorthalidone (1395058); HCTZ (974166); Indapamide (978555); Metolazone (907013)
Angiotensin converting enzyme inhibitors	Benazepril (1335471); Captopril (1340128); Enalapril (1341927); Fosinopril (1363749); Lisinopril (1308216); Moexipril (1310756); Perindopril (1373225); Quinapril (1331235); Ramipril (1334456); Trandolapril (1342439)
Angiotensin receptor blockers	Azilsartan (40235485); Candesartan (1351557); Eprosartan (1346686); Irbesartan (1347384); Losartan (1367500); Olmesartan (40226742); Telmisartan (1317640); Valsartan (1308842)
Dihydropyridine calcium channel blockers	Amlodipine (1332418); Felodipine (1353776); Isradipine (1326012); Nicardipine (1318137); Nifedipine (1318853); Nisoldipine (1319880)
Non-dihydropyridine calcium channel blockers	Diltiazem (1328165); Verapamil (1307863)
Beta blocker	Acebutolol (1319998); Atenolol (1314002); Betaxolol (1322081); Bisoprolol (1338005); Carvedilol (1346823); Labetalol (1386957); Metoprolol (1307046); Nadolol (1313200); Nebivolol (1314577); Penbutolol (1327978); Pindolol (1345858); Propranolol (1353766)
Alpha blocker	Doxazosin (1363053); Prazosin (1350489); Terazosin (1341238)
Other diuretics	Amiloride (991382); Bumetanide (932745); Eplerenone (1309799); Furosemide (956874); Spironolactone (970250); Torsemide (942350); Triamterene (904542)
Other drugs	Aliskiren (1317967); Clonidine (1398937); Guanfacin (1344965); Hydralazin (1373928); Methyldopa (1305447); Minoxidil (1307046)

© 2025 Lu Y et al. *JAMA Network Open.*

eTable 3. Adverse Cardiovascular Outcomes With Corresponding OMOP Concept IDs

**Term**	**OMOP Concept ID**
Myocardial infarction: Records from inpatient or ER visit only; Successive records with > 180-day gap are considered independent episodes	312327 (Acute myocardial infarction)<br>434376 (Acute myocardial infarction of anterior wall)<br>438438 (Acute myocardial infarction of anterolateral wall)<br>438170 (Acute myocardial infarction of inferior wall)<br>441579 (Acute myocardial infarction of inferoposterior wall)<br>436706 (Acute myocardial infarction of lateral wall)<br>4270024 (Acute non-ST segment elevation myocardial infarction)<br>4296653 (Acute ST segment elevation myocardial infarction)<br>46270162 (Acute ST segment elevation myocardial infarction due to left coronary artery occlusion)<br>46270163 (Acute ST segment elevation myocardial infarction due to right coronary artery occlusion)<br>43020460 (Acute ST segment elevation myocardial infarction involving left anterior descending coronary artery)<br>444406 (Acute subendocardial infarction)<br>4329847 (Myocardial infarction)<br>37309626 (Myocardial infarction due to demand ischemia)<br>4108677 (Subsequent myocardial infarction of anterior wall)<br>4108218 (Subsequent myocardial infarction of inferior wall)<br>45766241 (Subsequent non-ST segment elevation myocardial infarction)<br>45766114 (Subsequent ST segment elevation myocardial infarction)<br>439693 (True posterior myocardial infarction)
Ischemic stroke: Records from inpatient or ER visit only; Successive records with > 180-day gap are considered independent episodes	443454 (Cerebral infarction)<br>4110189 (Cerebral infarct due to thrombosis of precerebral arteries)<br>4111714 (Cerebral infarction due to cerebral venous thrombosis, non-pyogenic)<br>4108356 (Cerebral infarction due to embolism of cerebral arteries)<br>45772786 (Cerebral infarction due to embolism of middle cerebral artery)<br>4110190 (Cerebral infarction due to embolism of precerebral arteries)<br>46273649 (Cerebral infarction due to occlusion of basilar artery)<br>46270031 (Cerebral infarction due to occlusion of precerebral artery)<br>4110192 (Cerebral infarction due to thrombosis of cerebral arteries)<br>45767658 (Cerebral infarction due to thrombosis of middle cerebral artery)<br>40479572 (Infarct of cerebrum due to iatrogenic cerebrovascular accident)<br>4043731 (Infarction - precerebral)<br>4046360 (Lacunar infarction)<br>443864 (Multi-infarct dementia with depression)<br>4045737 (Pure motor lacunar infarction)<br>4045738 (Pure sensory lacunar infarction)

© 2025 Lu Y et al. *JAMA Network Open.*

Heart failure:<br>Records from inpatient or ER visit only; Successive records with > 180-day gap are considered independent episodes	44782718 (Acute combined systolic and diastolic heart failure)<br>312927 (Acute cor pulmonale)<br>40481042 (Acute diastolic heart failure)<br>44782733 (Acute on chronic combined systolic and diastolic heart failure)<br>40481043 (Acute on chronic diastolic heart failure)<br>37309625 (Acute on chronic right-sided congestive heart failure)<br>40480602 (Acute on chronic systolic heart failure)<br>4233424 (Acute right-sided heart failure)<br>40480603 (Acute systolic heart failure)<br>4242669 (Biventricular congestive heart failure)<br>4233224 (Cardiac insufficiency during AND/OR resulting from a procedure)<br>4264636 (Cardiac insufficiency following cardiac surgery)<br>44782719 (Chronic combined systolic and diastolic heart failure)<br>4229440 (Chronic congestive heart failure)<br>4195892 (Chronic cor pulmonale)<br>40479576 (Chronic diastolic heart failure)<br>4014159 (Chronic right-sided heart failure)<br>40479192 (Chronic systolic heart failure)<br>319835 (Congestive heart failure)<br>443587 (Diastolic heart failure)<br>316139 (Heart failure)<br>4004279 (High output heart failure)<br>44782728 (Hypertensive heart AND chronic kidney disease with congestive heart failure)<br>439696 (Hypertensive heart and renal disease with (congestive) heart failure)<br>439694 (Hypertensive heart and renal disease with both (congestive) heart failure and renal failure)<br>314378 (Hypertensive heart disease with congestive heart failure)<br>444101 (Hypertensive heart failure)<br>439846 (Left heart failure)<br>316994 (Malignant hypertensive heart disease with congestive heart failure)<br>4172864 (Neonatal cardiac failure)<br>4195785 (Right heart failure secondary to left heart failure)<br>4273632 (Right ventricular failure)<br>35615055 (Saddle embolus of pulmonary artery with acute cor pulmonale)<br>443580 (Systolic heart failure)

© 2025 Lu Y et al. *JAMA Network Open.*

eTable 4. Temporal Delay in Hypertension Diagnosis by Demographic Subgroups

Variables	Diagnosed after the second BP elevation (n = 45,454)	
	**Delayed diagnosis (month, median [IQR])**	**P-value (H-index, df)**
**Sex**		
Female	16.6 [5.8, 33.7]	<0.001 (199.0, 1)
Male	16.1 [5.7, 33.1]	
**Race/Ethnicity**		<0.001 (499.0, 4)
Non-Hispanic Asian	18.5 [6.9, 34.0]	
Non-Hispanic Black	17.2 [5.8, 34.9]	
Non-Hispanic White	16.3 [5.9, 33.3]	
Hispanic/Latinos	16.6 [5.6, 31.2]	
Others/Unknown	15.3 [4.9, 34.5]	
**Age Groups**		<0.001 (399.0, 3)
18-44	16.6 [5.6, 34.3]	
45-64	17.5 [6.1, 34.6]	
65-74	16.4 [5.9, 33.6]	
≥75	13.4 [4.7, 28.2]	

H-index was calculated from Kruskal-Wallis test; Df: degree of freedom

© 2025 Lu Y et al. *JAMA Network Open.*

eTable 5. Results of Cochran-Armitage Trend Test on Antihypertensive Medication Use According to Different Diagnosis Timing in Subgroups

Subgroups	Z statistics	P-value
Overall	-147.0	<0.001
Age groups		<0.001
Age 18-44	-71.5	<0.001
Age 45-64	-108.0	<0.001
Age 65-74	-61.8	<0.001
Age ≥75	-36.6	<0.001
Sex groups		<0.001
Female	-105.0	<0.001
Male	-102.0	<0.001
Race and Ethnicity		<0.001
Non-Hispanic Asian	-22.7	<0.001
Non-Hispanic Black	-74.2	<0.001
Non-Hispanic White	-120.0	<0.001
Hispanic/Latino	-22.8	<0.001
Others	-19.9	<0.001

© 2025 Lu Y et al. *JAMA Network Open.*

eFigure 1. Diagram Illustrating Categories of Diagnostic Timing

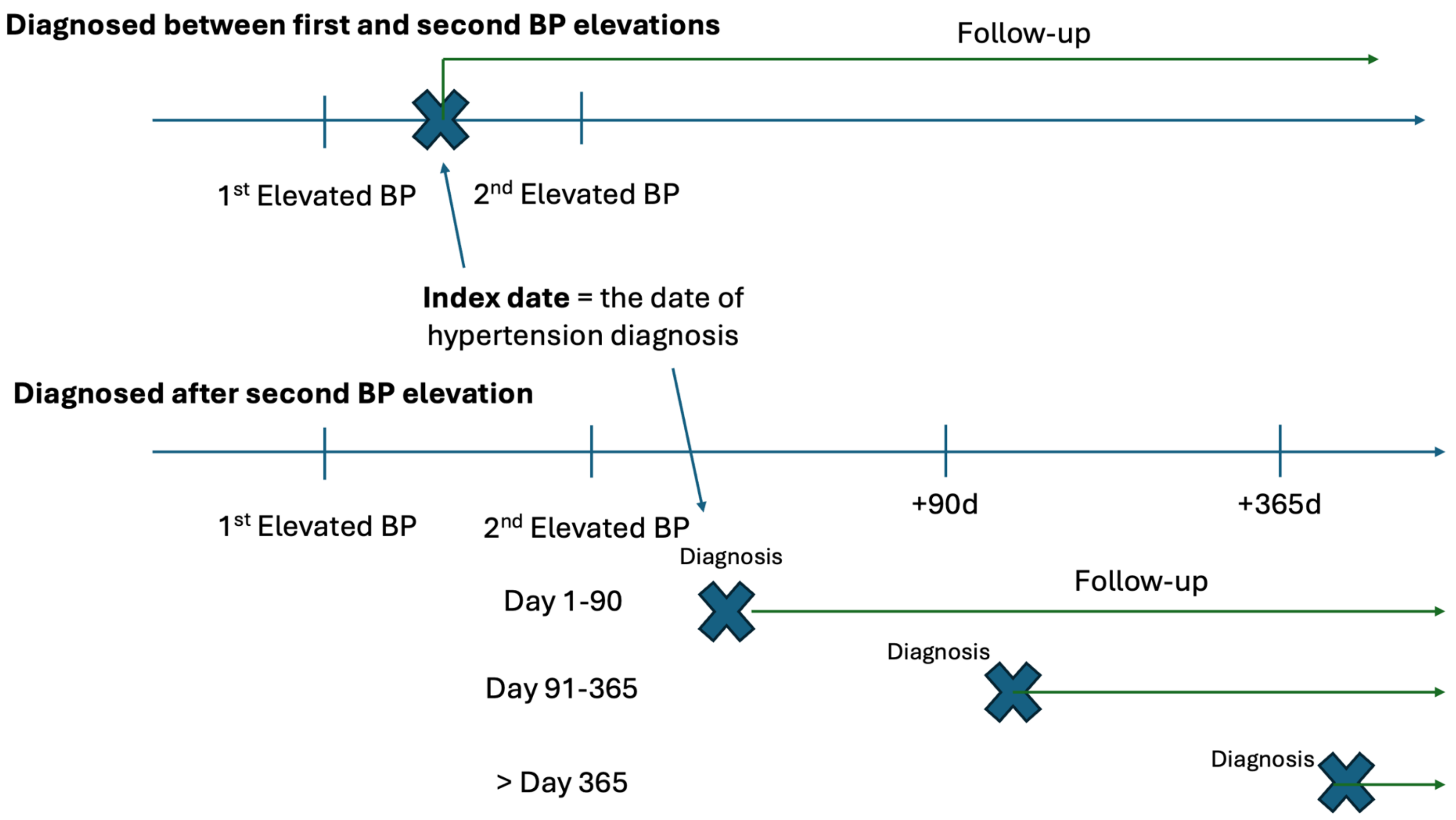

© 2025 Lu Y et al. *JAMA Network Open.*

eFigure 2. Study Flow Chart

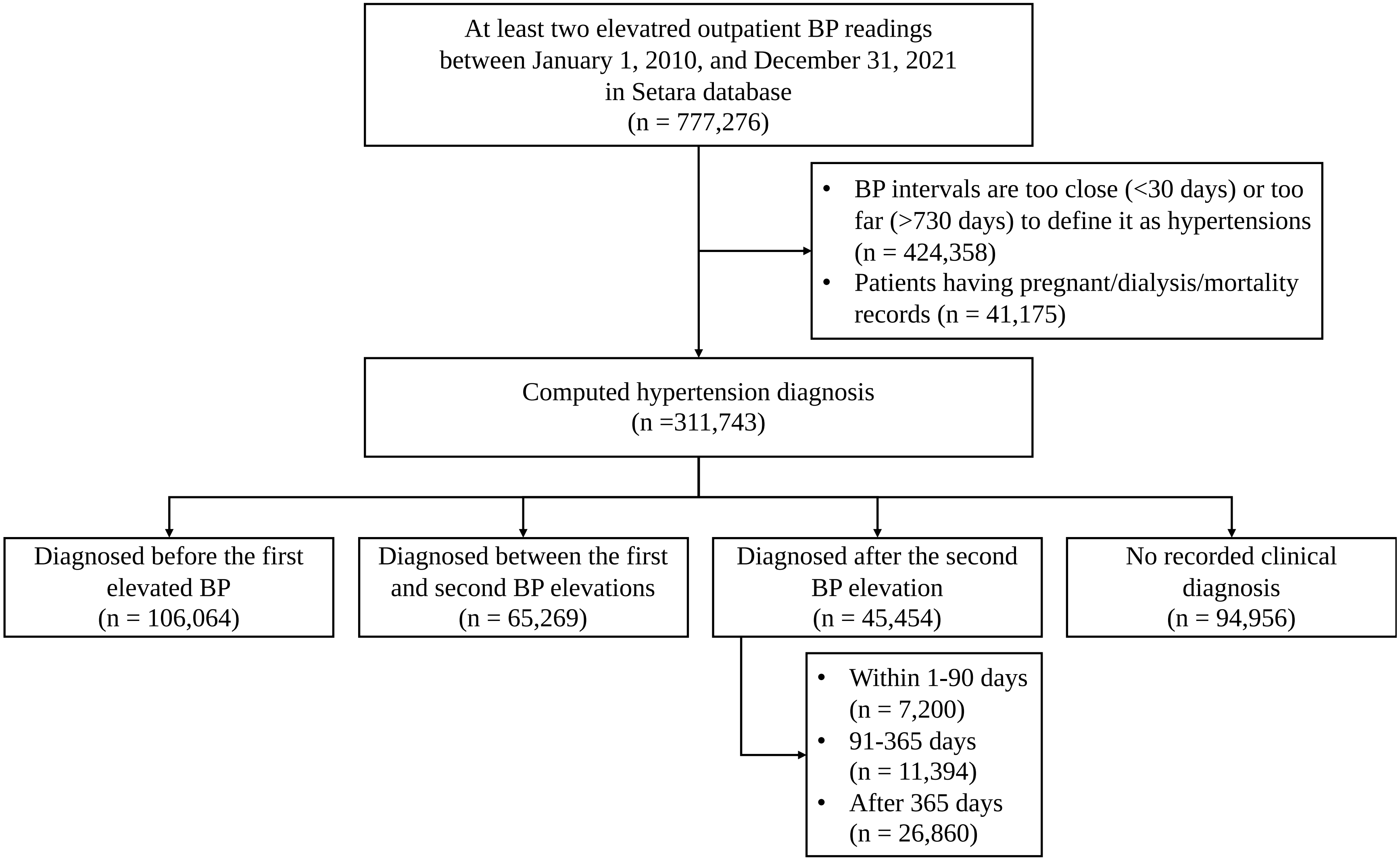

© 2025 Lu Y et al. *JAMA Network Open.*

eFigure 3. Prescription Rates of First-Line Antihypertensive Agents by Timing of Hypertension Diagnosis

(A) Overall (B) By age group (C) By sex (D) By race and ethnicity

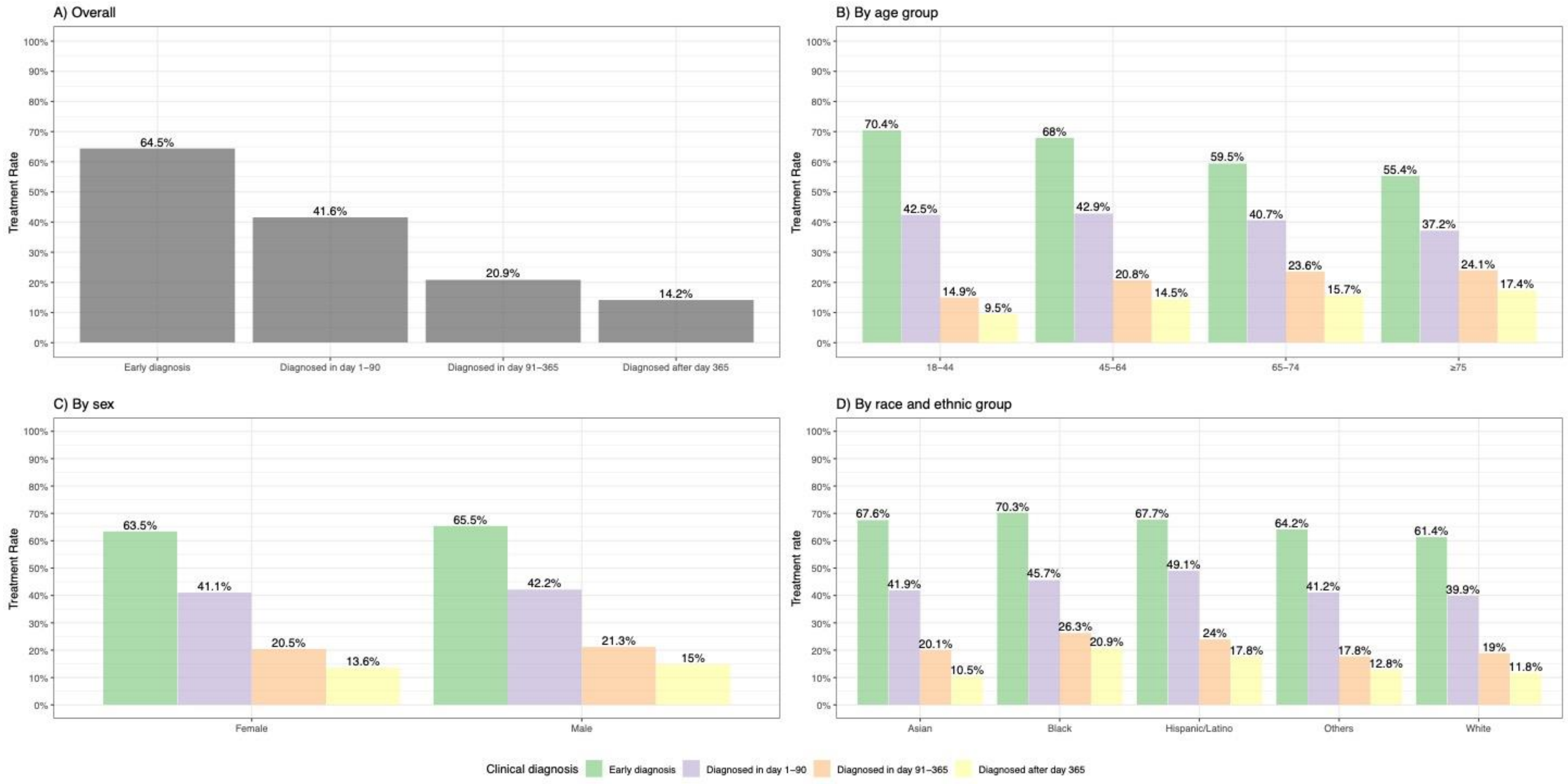

© 2025 Lu Y et al. *JAMA Network Open.*

eFigure 4. Age-Adjusted Survival Curves for Adverse Cardiovascular Outcomes Over a 5-Year Period by Age, Sex, Race, and Ethnicity

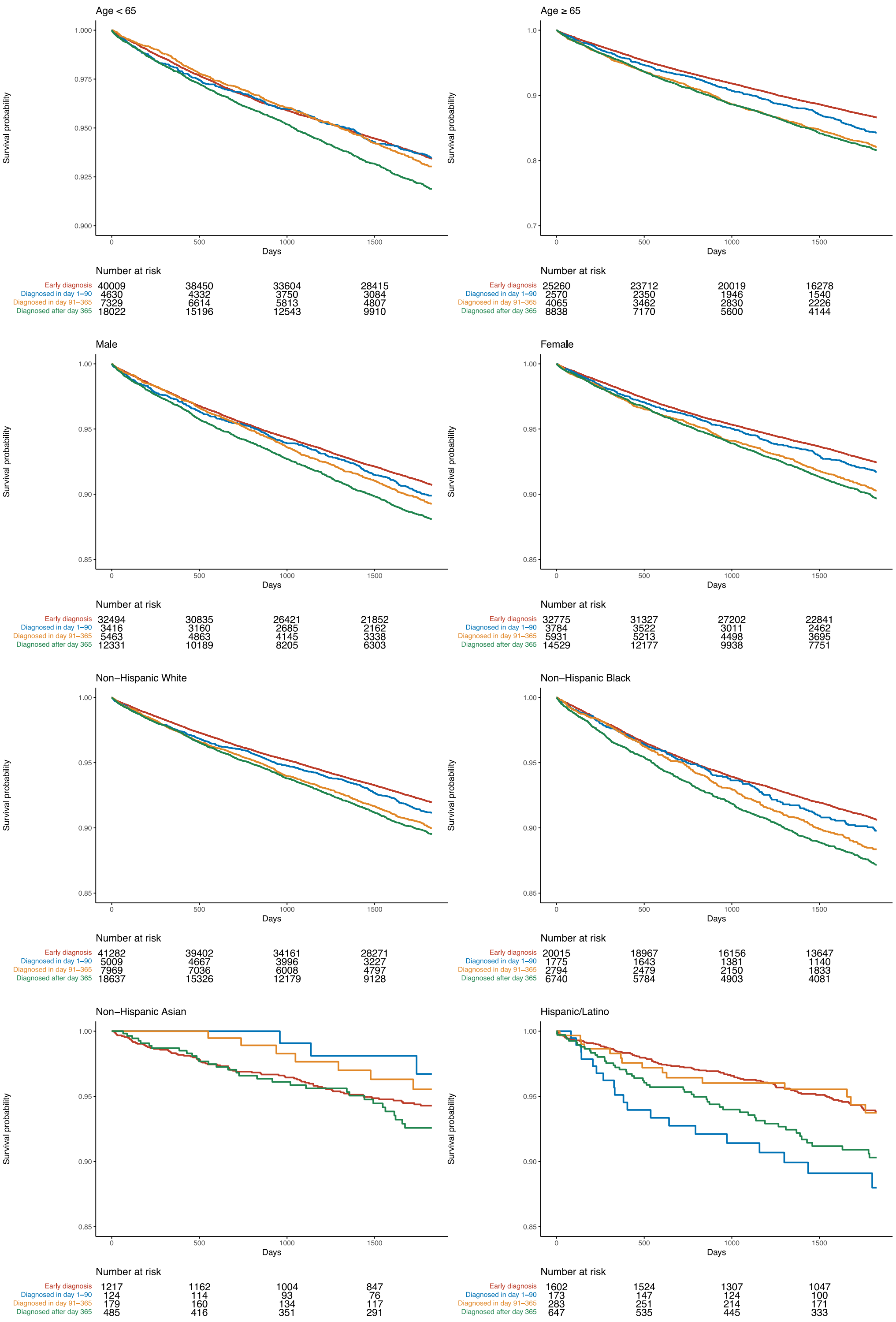

Footnote: The unexpected pattern observed in the Hispanic/Latino subgroup should be interpreted with caution due to the relatively small sample size in this group.
