# Supplementary material for: Delayed Hypertension Diagnosis and Its Association With Cardiovascular Treatment and Outcomes
Source: JAMA Netw Open. 2025 Jul 14;8(7):e2520498. doi: 10.1001/jamanetworkopen.2025.20498 (PMC12261005; doi:10.1001/jamanetworkopen.2025.20498)
Supplement: Supplement 2. — Data Sharing Statement [file jamanetwopen-e2520498-s002.pdf]

Data Sharing Statement

Lu. Delayed Hypertension Diagnosis and Its Association with Cardiovascular Treatment and Outcomes. *JAMA Netw Open*. Published July 14, 2025.
doi:10.1001/jamanetworkopen.2025.20498

Data
Data available: Data were obtained from a proprietary electronic health record database and are not publicly available. Deidentified individual participant data will not be shared. The study protocol and analytic code are available upon reasonable request from the corresponding author for academic purposes, contingent on approval by the institutional review board and data use agreements with the data provider.
